# Identification of *Uranotaenia sapphirina* as a specialist of annelids broadens known mosquito host use patterns

**DOI:** 10.1038/s42003-018-0096-5

**Published:** 2018-07-12

**Authors:** Lawrence E. Reeves, Chris J. Holderman, Erik M. Blosser, Jennifer L. Gillett-Kaufman, Akito Y. Kawahara, Phillip E. Kaufman, Nathan D. Burkett-Cadena

**Affiliations:** 1 Entomology and Nematology Department, Institute of Food and Agricultural Sciences, University of Florida, Gainesville, FL 32611 USA; 2 Florida Medical Entomology Laboratory, Institute of Food and Agricultural Sciences, University of Florida, Vero Beach, FL 32962 USA; 3 Florida Museum of Natural History, University of Florida, Gainesville, FL 32611 USA; 4 Present Address: Florida Medical Entomology Laboratory, Institute of Food and Agricultural Sciences, University of Florida, Vero Beach, FL 32962 USA

## Abstract

Feeding upon vertebrate blood by mosquitoes permits transmission of diverse pathogens, including viruses, protozoa, and nematodes. Despite over a century of intensive study, no mosquito species is known to specialize on non-vertebrate hosts. Using molecular analyses and field observations, we provide the first evidence, to our knowledge, that a mosquito, *Uranotaenia sapphirina*, specializes on annelid hosts (earthworms and leeches) while its sympatric congener, *Uranotaenia lowii*, feeds only on anurans (frogs and toads). Our results demonstrate that *Ur. sapphirina* feeds on annelid hosts (100% of identified blood meals; *n* = 72; collected throughout Florida), findings that are supported by field observations of these mosquitoes feeding on *Sparganophilus* worms and freshwater leeches. These findings indicate that adult mosquitoes utilize a much broader range of host taxa than previously recognized, with implications for epidemiology and the evolution of host use patterns in mosquitoes.

## Abstract (web summary)

Lawrence Reeves et al. report evidence that adult females of the mosquito species *Uranotaenia sapphirina* feed primarily on annelid hosts. This is the first known example of a mosquito species that specializes on invertebrate blood and suggests that mosquito host use patterns are more diverse than previously recognized.

## Introduction

Mosquitoes (Diptera: Culicidae) are the vectors of disease-causing human and wildlife pathogens^1–8^, and as a result, they have received greater scientific and public attention than any other insect taxon. The evolutionary innovation of blood feeding in the nematoceran flies preceded our species by hundreds of millions of years^9^, but has had persistent consequences throughout human history. Because blood feeding enables the transmission of pathogens between vertebrate hosts^10^, it is the primary reason behind the intense study of mosquitoes and their interactions with vertebrates. Despite this, the origin of blood feeding and the evolution of host use patterns in mosquitoes or other dipteran vectors of pathogens remains poorly understood. Blood feeding presents an evolutionary challenge that requires meticulous adaptations that are specific for various host groups. To effectively utilize blood, a mosquito must not only possess olfactory, visual, or thermal machinery to locate hosts and pierce the epidermis, but overcome cellular and molecular barriers to blood feeding^11^ that vary between host orders^12^. Most vertebrate animals have evolved complex cellular mechanisms that rapidly respond to blood vessel injuries with a series of immune and hemostatic reactions. Mosquitoes that feed on endothermic hosts (birds and mammals) must also possess thermoregulatory strategies to avoid overheating^13^. The evolution of mechanisms around these barriers in mosquitoes, including biochemical salivary cocktails that circumvent immune and hemostatic responses of hosts, and thermoregulation and highly specialized mouthparts and sensory organs, have had tremendous implications for human health, throughout history and today.

Feeding on vertebrate blood is characteristic of mosquitoes of all genera, with few exceptions. All species of *Toxorhynchites* (89 species) and *Malaya* (12 species), and possibly others (e.g., *Topomyia*, *Maorigoeldia*) do not require a blood meal to complete egg development^14–18^. Adults of species of these genera feed exclusively on plant-derived sugars, either directly^15^ or from the carbohydrate-rich solution regurgitated by ants^18^. Autogenous, or partially autogenous, mosquito species are also found in a number of genera which include species that are otherwise blood-feeders^19–23^. Many blood-feeding mosquito species feed on plant-derived sugars to support metabolism^10^, but only females feed on blood. Female mosquitoes of hematophagous species feed on diverse vertebrate lineages, including mammals, birds, reptiles, amphibians, and fishes^24^. Most specialize to varying degrees on certain ranges of vertebrate classes or orders, and these patterns of host use mediate the transmission dynamics of mosquito-vectored pathogens^25^. No mosquito species studied to date has been found to specialize on the blood of a non-vertebrate animal.

Mitigating the impact of vector-borne pathogens is one of the greatest challenges in epidemiology and medicine. Because the transmission networks of mosquito-vectored pathogens are structured by mosquito host use patterns, understanding mosquito–host interactions is a critical element in confronting this challenge. Blood meal analysis is a collection of techniques that takes advantage of immunological or genetic specificity of host blood in adult mosquito gut contents in order to identify hosts^24^. Using PCR-based blood meal analyses, we investigated the host use patterns of the two *Uranotaenia* species that occur in eastern North America: *Ur. sapphirina* and *Ur. lowii*.

*Uranotaenia* is a taxonomically diverse genus, consisting of 270 currently recognized, primarily tropical species^14, 26^. Although few species have been extensively studied, those investigated feed primarily on anuran hosts^27, 28^ and at least one feeds on amphibious fishes^10^. *Uranotaenia lowii* feeds predominantly on frogs, and host-seeking females are attracted to their songs^29^. Until now, the host use patterns of *Ur. sapphirina* were, as far as we are aware, unknown, and despite deficient evidence, assumed to parallel those of *Ur. lowii*. Previous research using both serological and DNA-based blood meal analyses have attempted to identify the hosts of *Ur. sapphirina*, but most blood meal assays failed. For example, Irby and Apperson^30^ identified only two (1.7%) of 120 *Ur. sapphirina* blood meals (both as an unknown species of snake) and Cupp et al.^31^ identified 2 (5.7%) of 35 (both as the ranid frog *Lithobates catesbeianus*), compared with identification rates of 85.8% and 61.4%, respectively, for all mosquito species screened, excluding *Ur. sapphirina*.

We determined that *Ur. sapphirina* is a specialist of invertebrate hosts, worms, and leeches of the phylum Annelida, while the sympatric *Ur. lowii* specializes on the amphibian order Anura (frogs). We demonstrate that adult *Ur. sapphirina* feed on diverse annelid hosts, and report the first documentation, to our knowledge, of a mosquito specializing on invertebrate hosts. We collected blood-fed *Uranotaenia* mosquitoes (*n* = 132; 88 *Ur. sapphirina*; 44 *Ur. lowii*) from multiple locations on the Florida Peninsula. We used two diagnostic PCR assays to screen *Uranotaenia* blood meals for annelid and vertebrate DNA. Annelid DNA was targeted because field observations made at River Styx, Alachua Co., Florida, USA, suggested that this mosquito was feeding on oligochaete earthworm hosts. For each blood meal, we used extracted DNA as amplification templates in two separate reactions: one that targeted the annelid *28S ribosomal RNA* and one that targeted the vertebrate *cytochrome* *c* *oxidase subunit I gene* (*COI*). Amplification reactions and primers were designed to produce an amplicon only in the presence of their respective template. Products of all successful reactions were sequenced to confirm the presence of annelid or vertebrate DNA.

## Results

### Mosquito collections

In total, 132 blood-fed adult female *Uranotaenia* mosquitoes were collected in four counties in Florida, representing 88 *Ur. sapphirina* and 44 *Ur. lowii*. *Uranotaenia sapphirina* was collected in Columbia Co. (*n* = 18), Alachua Co. (*n* = 14), and Indian River Co. (*n* = 56). *Uranotaenia lowii* was collected in Alachua Co. (*n* = 26), Indian River Co. (*n* = 14), and Miami-Dade Co. (*n* = 4).

### Host identification

**Fig. 1:**
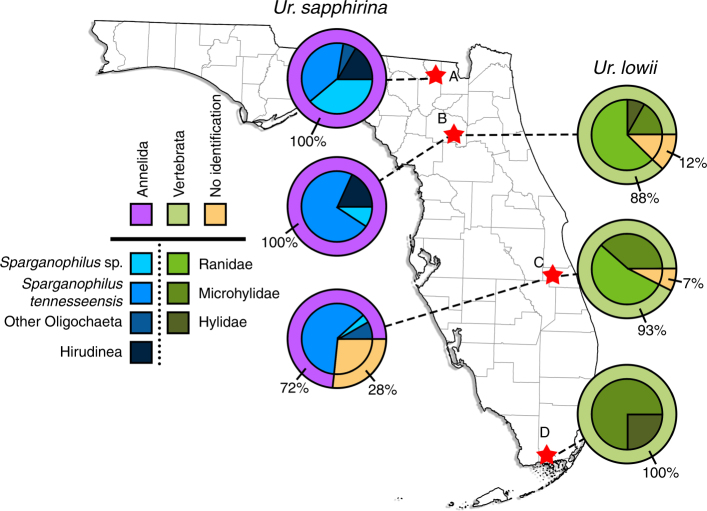
Host use patterns of *Ur. sapphirina* and *Ur. Lowii*. Mosquitoes were collected in four counties (A–D) along the Florida Peninsula, USA, and host use patterns were determined by diagnostic PCR screening of blood meals for annelid and vertebrate DNA. Results of PCR screens for vertebrate and annelid DNA in the blood meals of 88 *Ur. sapphirina* (left) and 44 *Ur. lowii* (right), collected from Columbia Co. (A) Alachua Co. (B), Indian River Co. (C), or Miami-Dade Co. (D). Pink shading of outer rings indicates the proportion of blood meals that screened positive for annelid DNA and were confirmed by Sanger sequencing. Green shading on outer rings indicates the proportion of blood meals that screened positive for vertebrate DNA and were confirmed by Sanger sequencing. Orange shading on outer rings and inner circles indicates the proportion of blood meals that either did not produce an amplicon or resulted in ambiguous sequences that could not be attributed to a host taxon. Blue shading of inner circles represents the proportion of blood meals derived from various annelid taxa, as determined by DNA sequences. Green shading of inner circles represents the proportion of blood meals derived from various vertebrate taxa, as determined by DNA sequences. Excluding unidentified blood meals (orange shading), the proportion of annelid and vertebrate hosts overall was significantly different between *Ur. sapphirina* and *Ur. lowii* (two-sided Fisher’s Exact Test; *P* *<* 0.001). Map created using data obtained from the Florida Geographic Data Library^78^

The results of PCR assays indicated that *Ur. sapphirina* and *Ur. lowii* had distinct and disparate host specialization patterns (Fig. 1). There was a significant difference in the use of annelid and vertebrate hosts between *Ur. sapphirina* and *Ur. lowii* (two-sided Fisher’s exact test; *P* *<* 0.001). We found that 100% of identified *Ur. sapphirina* blood meal DNA was derived from annelid hosts, while 100% of identified *Ur. lowii* blood meal DNA was derived from anuran hosts. Templates from 80 of 88 *Ur. sapphirina* blood meals screened positive for annelid DNA, and of these, 72 (81%) were confirmed by Sanger sequencing. All *Ur. sapphirina* blood meals were negative for vertebrate DNA. Identical screens of *Ur. lowii* blood meals indicated that 43 of 44 were positive for vertebrate DNA, with 38 (86%) confirmed by Sanger sequencing and attributed to anuran species known to occur in Florida. All *Ur. lowii* blood meals were negative for annelid DNA. Recovered host DNA sequences were compared against a reference database (GenBank, National Center for Biotechnology Information), or sequences obtained from morphologically identified annelid specimens. The majority (93%) of identified *Ur. sapphirina* blood meals were attributed to oligochaete earthworms. *Sparganophilus tennesseensis*, a sparganophilid worm, was the most frequently identified host, detected in 43 of 72 (60%) of *Ur. sapphirina* blood meals. Two species of freshwater leeches (*Macrobdella ditetra*, *Philobdella floridana*) together represented 7% of identified *Ur. sapphirina* blood meals. In comparison, the hosts of *Ur. lowii* were exclusively identified as anurans.

### Field observations

**Fig. 2:**
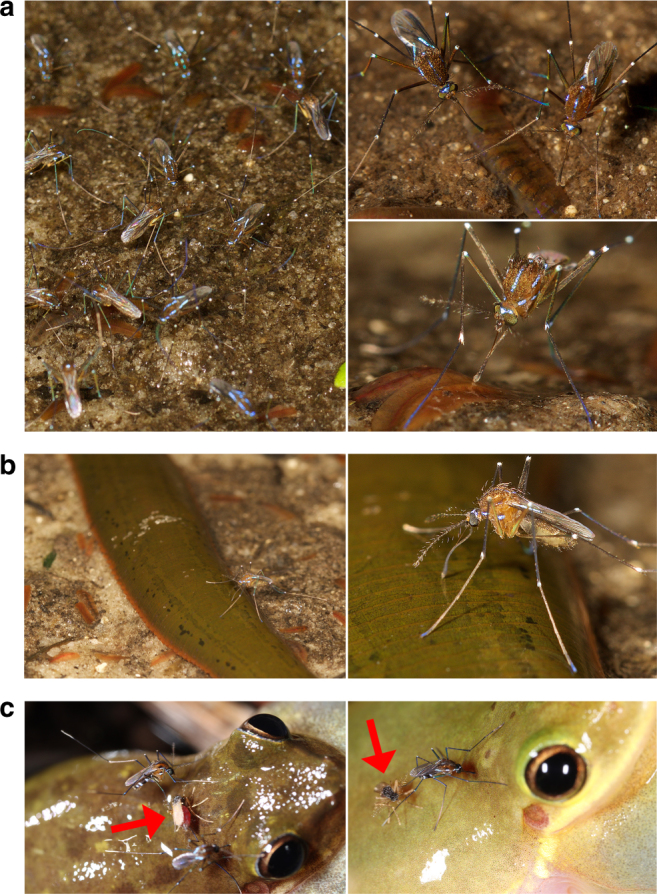
Interactions between *Uranotaenia*, *Corethrella,* and hosts. **a**, **b** Female *Uranotaenia sapphirina* were observed, photographed, and filmed at River Styx, Alachua Co., Florida, USA. **a** Congregations of female mosquitoes were observed questing among and feeding from partially submerged *Sparganophilus* earthworms. **b** Female *Uranotaenia sapphirina* feeding from the leech *Macrobdella ditetra*; note reflexed labium and exposed stylets. **c** Females of *Uranotaenia lowii* and frog-biting midges (Corethrellidae; indicated by red arrows) feeding from *Hyla squirella*, Miami-Dade Co., Florida, USA

To further confirm these results, we made field observations of *Ur. sapphirina* and *Ur. lowii* in Alachua Co., Florida. We observed and documented female *Ur. sapphirina* feeding on both oligochaete worms and freshwater leeches (Fig. 2) at the interface between terrestrial and aquatic ecosystems. Female *Ur. sapphirina* were observed probing the substrate with their proboscises, presumably in attempts to locate hosts (Supplementary Movie 1). While no *Ur. lowii* were observed feeding on annelids, we documented females feeding on both hylid and ranid frogs (Fig. 2).

## Discussion

Unlike any previously studied mosquito species, *Ur. sapphirina* in our sample fed exclusively on annelid hosts. This finding explains the inability of previous investigations to identify *Ur. sapphirina* blood meals, as these studies were performed under the assumption, and corresponding laboratory methodology, that female mosquitoes take blood meals only from vertebrate animals. Annelids and vertebrates share enclosed circulatory systems and either extracellular (annelids) or intracellular (vertebrates) hemoglobin, which in both groups causes the characteristic red coloration of the blood. The presence of red blood in the guts of *Ur. sapphirina* females likely contributed to the confusion related to host use of this mosquito by other researchers. Future studies may need to consider the possibility that mosquitoes fed on other types of invertebrates may not display the red gut normally used to classify a mosquito as blood engorged.

The recognition of *Ur. sapphirina* as a specialist of annelid, not vertebrate, host animals has important implications in mosquito ecology and evolution, and in the epidemiology of mosquito-vectored pathogens. This finding demonstrates that the range of potential mosquito hosts is considerably broader than previously indicated. *Uranotaenia sapphirina* is a common species throughout eastern North America, where mosquitoes have been under extensive study since their involvement in pathogen transmission was recognized in 1881 (ref. ^1^). The presumption that adult female mosquitoes blood feed only from vertebrate hosts^10^ is a source of bias in the methodological framework used to study mosquito ecology. Mosquito blood meals are identified primarily through methods that, by design, selectively react with only vertebrate antigens or DNA. For this reason, estimating the extent to which invertebrate hosts are utilized by female mosquitoes is not possible given previously available methods. In the laboratory, caged mosquitoes, including mammalophilic *Aedes* and *Anopheles* species have been documented locating and feeding on lepidopteran larvae in no-choice experiments, and subsequently, in some cases, to produce viable eggs^32–35^. Anecdotal records of mosquitoes feeding on cicada nymphs, mantids, chironomid midges, and lepidopteran pupae reported in the early 1900s by Howard^36, 37^ and occasionally referenced in the literature^26, 38^ have been disputed by Downes^10^ as mistaken identifications and include few substantive details. Beyond these laboratory experiments and historic records, there is no previous evidence that suggests that such interactions occur in nature, although any instance of invertebrate host use would not be detected by the traditional methods of blood meal analysis. Interestingly, for some mosquito and Corethrellidae (the culicomorph sister taxon to mosquitoes + Chaoboridae^39^) species, blood meal analyses that are effective in other species have failed, which may indicate that these species also feed upon hosts that cannot be detected using the vertebrate-based methodology^40–43^. As new sequencing technologies are applied to blood meal analysis, the ability to detect unexpected hosts should improve, particularly with the recognition of the potential for invertebrate feeding in mosquitoes.

Understanding the extent to which mosquitoes, particularly pathogen vectors, interact with invertebrate hosts has epidemiological implications. *Uranotaenia sapphirina* has been implicated as a potential vector for several arboviruses. Field-collected *Ur. sapphirina* have tested positive for Eastern equine encephalitis virus^2, 4, 44, 45^ and West Nile virus^3^. Our results suggest that *Ur. sapphirina* is unlikely to become infected with these viruses through feeding on vertebrate hosts. It is possible that by feeding on hematophagous leeches, which themselves often parasitize competent arbovirus hosts^46, 47^, *Ur. sapphirina* could act as kleptoparasites, acquiring virus-infected vertebrate blood meals from their leech hosts. Similarly, interactions between *Ur. sapphirina* and leeches may affect the transmission of pathogens vectored by leeches^48^. In previous studies^30, 31^, a small proportion of examined *Ur. sapphirina* blood meals were derived from snakes and the ranid frog *Lithobates catesbeianus*. Some snake species (e.g., *Agkistrodon piscivorus*, *Nerodia* spp.) and ranid frogs are common at the margins of vegetated waterways at night, a microhabitat where *Ur. sapphirina* females were observed feeding on annelid hosts. In these microhabitats, snakes and frogs would be available to host-seeking *Ur. sapphirina* females, and may be fed on incidentally. An alternative possibility for the detection of arboviruses in *Ur. sapphirina* is that this mosquito occasionally feeds on vertebrate hosts that may be competent for some arboviruses. The identification of two snake-derived *Ur. sapphirina* blood meals^30^ is particularly noteworthy, as the role of snakes in arbovirus persistence and transmission has been increasingly supported^31, 49–52^. Future studies should elucidate how *Ur. sapphirina* comes into contact with these viruses, and investigate the possibility that *Ur. sapphirina* could represent a complex of cryptic species with varying host use patterns.

Annelid host specialization by *Ur. sapphirina* raises important questions about the origin and evolution of blood feeding in mosquitoes. Foremost is whether feeding on invertebrates is an ancestral or derived trait. Divergence and radiation time estimates of Culicomorpha and vertebrate host lineages suggest that mosquitoes, or their culicomorph ancestors, adapted to vertebrate host groups after their diversification. The relationship between host and mosquito/Culicomorpha phylogenies has yet to be assessed, but other hematophagous insects have undergone stepwise transitions, with diversification of hematophagous insect lineages paralleling their host phylogenies^53^. Understanding how invertebrates factor into the evolution of host use by mosquitoes and other Culicomorpha will ultimately depend on a more complete accounting of mosquito host use patterns and the extent of invertebrate host use, and a well-resolved mosquito phylogeny. Until more information becomes available, understanding the origins and evolution of blood feeding in mosquitoes will remain speculative. However, the evolutionary history of Culicomorpha and host animals, and the host use patterns of basal mosquitoes may provide clues.

**Fig. 3:**
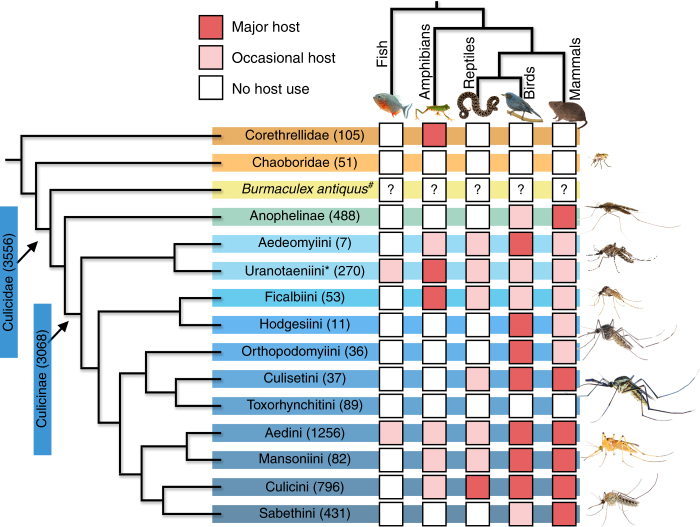
Patterns of blood-feeding on vertebrate host classes for Culicidae and related lineages (Corethrellidae and Chaoboridae). Composite image adapted using phylogeny from Harbach and Kitching^61^ and Borkent and Grimaldi^54^. Numbers in parentheses following mosquito taxa indicate number of species within each taxon^14, 57, 79^. Shading of squares indicates the degree to which mosquito or related lineages interact with vertebrate classes^14, 15, 24, 27, 28, 30, 31, 40, 43, 54, 57, 62, 79–95^. *Uranotaenia sapphirina* is contained within tribe Uranotaeniini*. All mosquito lineages are extant except *Burmaculex antiquus*^#^, the oldest known fossil mosquito, from the middle Cretaceous (110 mya). *Burmaculex antiquus* is presumed to have been a blood feeder, but had a moderately elongate proboscis and lacked the palpal sensillae that function as CO_2_ receptors for host location in modern mosquitoes^54^

Birds and mammals are the major hosts of many modern mosquitoes, particularly among the more derived lineages (Fig. 3). However, it is unlikely that birds or mammals were the initial hosts of ancestral mosquitoes, as the earliest known fossil mosquito, *Burmaculex antiquus*^54^, precedes the diversification of birds^55^ and mammals^56^ by 30–40 million years. Modern frog-biting midges (Corethrellidae), sister to the mosquitoes + phantom midges (Chaoboridae)^39^, are known to feed only on anuran hosts (Fig. 3), and this association dates to the Lower Cretaceous^57^, pre-dating *Burmaculex* by 75 million years. The antiquity of anuran host use by *Corethrella* and the use of endothermic hosts by modern mosquitoes suggests a relationship between the vertebrate and mosquito phylogenies. However, this hypothesis is not supported by the basal placement of *Anopheles*, the human malaria vectors, that are generally considered specialists of mammalian hosts (Fig. 3). The split between Anophelinae and Culicinae is estimated at 45–126 million years before *Burmaculex*^58, 59^, suggesting either that mammal specialization in Anophelinae is not the ancestral trait, or that the basal placement of Anophelinae is incorrect. While phylogenetic analyses based on molecular data have not yet fully resolved deeper (genus-level) divisions within Culicidae^26^, they have estimated the time of divergence of *Uranotaenia* from other genera at >150 mya^58^. This event would have been concurrent with the diversification of major anuran groups^60^ and the first actual fossils of frog-biting midges^57^, and 50 million years older than *Burmaculex*. *Uranotaenia*, with its sister group *Aedeomyia*, is placed in a basal position within Culicinae^58, 61^ (Fig. 3), a diverse clade containing the majority of mosquito genera, implying an ancient origin for *Uranotaenia*. This might suggest that, if invertebrate feeding is the pleisiomorphic state, host affinities within *Uranotaenia* are indicative of early patterns of host use within Culicidae that were lost in other basal lineages (e.g., Anophelinae).

The limited evidence available indicates that the earliest lineages of Culicomorpha fed on the body fluids (hemolymph) of the open circulatory systems of insects^62^. This trait is retained in multiple culicomorph families, including some extant chironomids and ceratopogonids, and mammalophilic *Anopheles*^33^ and *Aedes*^32, 34^ mosquitoes are able to locate insect hosts and utilize hemolymph to mature eggs. The closed circulatory systems of annelids, by contrast, are analogous to those of vertebrates, with phenotypically similar components, including heart(s), blood vessels, and hemoglobin^63, 64^. Annelid feeding may represent an evolutionary link between by an ancestral culicomorph feeding on free hemolymph in the body cavity and modern mosquitoes feeding on vertebrates with closed circulatory systems. While annelid circulatory systems are analogous to those of vertebrates, annelid immune systems and hemostatic responses are less sophisticated^65, 66^, suggesting that annelid feeding mosquitoes may encounter fewer defenses. In that context, annelid feeding may have primed the development of physiological, biochemical, behavioral, and morphological adaptations that would enable mosquitoes to eventually circumvent barriers to blood feeding from diverse vertebrate hosts, ultimately leading to the tremendous human health impacts caused by pathogen transmission in modern mosquitoes.

The use of annelid hosts in *Ur. sapphirina* alternatively could be a trait derived from frog feeding ancestors switching to worms and leeches that were encountered in habitats similar to anuran hosts. The phylogenetic position (Fig. 3) of the frog-biting midges, and their presumed ancient association with frogs^57^ may suggest that amphibians were the hosts of the common ancestor of Corethrellidae and Culicidae + Chaoboridae. However, a recent study of the hosts of *Corethrella* found that PCR assays successfully identified only <30% of blood meals, despite the use of primers that amplify a broad range of vertebrate hosts^43^. The authors of that study^43^ compared their low success rate with that of *Ur. sapphirina* from other published works, leading them to conclude that female *Corethrella* may feed on additional, undetermined hosts.

The function of annelid host use by *Ur. sapphirina* is not yet established, and the molecular analyses and field observations we report cannot discount the possibility that annelid host use by *Ur. sapphirina* could be a derived trait that evolved to serve a non-reproductive function, such as preventing dehydration or supplementing energetic reserves. Blood feeding by mosquitoes serves a function that is primarily reproductive: the females of most mosquito species require nutrients, particularly proteins, from host blood to provision developing eggs^10^, although blood-derived resources can also be diverted to meet metabolic needs^67, 68^ or in response to dehydration^69^. Dehydration can alter the behavior of mosquitoes by prompting them to increase host seeking and blood feeding^69^. Carbohydrates, obtained directly or indirectly from plants, serve primarily as a metabolic resource to both male and female mosquitoes, but can also enhance the reproductive potential of a female mosquito^70^. Our findings do not exclude the potential that female *Ur. sapphirina* utilize annelid blood as an energetic resource or a means of maintaining hydration; however, circumstantial evidence supports the idea that annelid feeding plays a reproductive role for this mosquito. For example, despite the collection of numerous male *Ur. sapphirina*, blood meals were only found in females in our samples. In addition, both males and females have been observed nectaring on flowers at field locations (L.E.R., personal observation), and collected engorged with nectar^71^. The details of egg production following annelid feeding, and the potential for annelid blood to serve a function other than egg maturation, needs to be explored by subsequent research to better understand the evolutionary context of these findings, as our data are limited to the identification of annelids as the hosts of *Ur. sapphirina*.

Specialization of *Ur. sapphirina* on annelid hosts demonstrates that the host breadth of mosquitoes is substantially broader than previously understood. Prior research on the host interactions of mosquitoes has centered around a minor subset of mosquito species, particularly the *Aedes*, *Anopheles*, and *Culex* pathogen vectors. For many genera, particularly those restricted to tropical regions, host use patterns have not been investigated, leaving substantial gaps in the understanding of mosquito–host relations. Combined with the large diversity of mosquitoes, there is potential that invertebrate host specialization extends beyond *Ur. sapphirina*. The fact that a common North American mosquito specializing on annelid hosts has gone undocumented as far as we are aware for more than a century suggests that invertebrate host use by mosquitoes is easily overlooked. Future work towards a more complete understanding of mosquito host use patterns should consider this possibility and, ideally, make use of novel molecular technologies that are compatible with the detection of invertebrate hosts.

## Methods

### Mosquito collections

Blood engorged *Uranotaenia* mosquitoes were collected between 28 September 2015 and 10 May 2017 at sites throughout the Florida Peninsula: Osceola National Forest (Columbia County), River Styx (Alachua County), Newnan’s Lake (Alachua County), the University of Florida Natural Area Teaching Lab (Alachua County), two sites at Blue Cypress Lake Conservation Area (Indian River County), and Everglades National Park (Miami-Dade County; Permit Number EVER-2017-SCI-0011). Mosquitoes were collected from natural resting sites (tree trunks, cypress knees, vegetation, exposed tree roots) using a battery-powered aspirator. Mosquitoes from Osceola National Forest, River Styx, Natural Area Teaching Lab, and Everglades National Park were killed in the field, promptly after collection, by exposure to ethyl-acetate-soaked plaster for approximately 10 min. Host DNA was immediately preserved in the field on Whatman Flinders Technology Associates (FTA) blood cards^72^, and taken to the Entomology and Nematology Department, University of Florida (Gainesville, Florida) for blood meal analysis. Mosquitoes from Indian River Co. were promptly transported to the laboratory (Florida Medical Entomology Laboratory, Vero Beach, Florida) inside polypropylene collecting cups (BioQuip), chilled on ice inside a cooler. Upon arrival, collecting cups were placed into a −20°C freezer for at least 20 min to kill mosquitoes. Mosquitoes were identified through morphological characters^73^. Blood-fed individuals were separated from others by visual inspection of the abdomen. DNA was extracted using the hot sodium hydroxide and tris (HotSHOT) method^74^ or DNEasy blood and tissue kit (Qiagen), following the manufacturer’s protocol.

### PCR and DNA amplification

For each blood meal DNA sample, we attempted to amplify template DNA fragments in two independent PCRs targeting vertebrate or annelid DNA, respectively. One PCR was intended to screen for vertebrate DNA and used a vertebrate-specific primer set that amplifies a 664 bp template of the *COI* barcode region^75^. The primers RepCOI-F and RepCOI-R (Table 1) were used in 20 µL reactions each consisting 10 µL, 2× Apex Taq RED Mastermix (Genesee Scientific), 0.75 µL each primer (10 μM), 7.5 µL molecular grade water, and 1 µL DNA template. Thermocycler conditions followed a profile of 94 °C for 3 min, followed by 40 cycles of 94 °C for 40 s, 48.5 °C for 30 s, and 72 °C for 1 min, and a final extension step of 72 °C for 7 min. The other PCR was intended to screen for annelid DNA and used an annelid-specific primer set to amplify a 416 bp fragment of the annelid *28S ribosomal RNA*. We used forward primer SPARG_C2_416 designed de novo to exclude co-amplification of *Uranotaenia* templates with reverse primer 28S_C4_R (Table 1)^76^. PCR reactions consisted of 12.5 μL Invitrogen^TM^ Platinum Green Hot Start PCR 2× Master Mix kit plus 0.5 µL each primer (20 μM), 2.5 µL DNA template, 9 µL molecular grade water, and followed a thermal profile of 94 °C for 2 min, followed by 35 cycles of 94 °C for 30 s, 59.3 °C for 30 s, and 72 °C for 1 min, followed by a final extension step of 72 °C for 1 min. Negative controls were included in each set of amplification reactions and utilized sterile, double-distilled water in place of extracted DNA to monitor for contamination. PCR products were stained with ethidium bromide or SYBR™ Safe DNA Gel Stain (Invitrogen^TM^) and visualized under ultra-violet or blue light after electrophoresis on a 1.0% or 1.5% agarose gel. DNA ladder, 50 or 100 bp (Invitrogen^TM^), was used to determine the approximate fragment size of PCR products.

**Table 1:** Primer sequences used to screen *Uranotaenia* blood meals for vertebrate and annelid DNA

Primer name	Target	Sequence	Amplicon size (bp)	Reference
RepCOI-F	Vertebrate *COI*	5′-TNT TMT CAA CNA ACC ACA AAG A-3′	664	^75^
RepCOI-R	Vertebrate *COI*	5′-ACT TCT GGR TGK CCA AAR AAT CA-3′	664	^75^
SPARG_C2_416_F	annelid *28S ribosomal RNA*	5′-ATC GGT CGG CAA CCT GTC CG-3′	416	This study
28S_C4_R	annelid *28S ribosomal RNA*	5′-TTC GAT TRG TCT TTC GCC CCT-3′	416	^76^

### DNA sequencing and taxonomic identification

To confirm that the PCRs correctly detected annelid or vertebrate DNA, any PCR product that showed a band at the expected fragment size was submitted to the University of Florida Interdisciplinary Center for Biotechnology Research or Eurofins for Sanger sequencing on an ABI 3130^®^ automated sequencer. Resulting sequence chromatograms were examined and edited for quality in the program Geneious^®^ Version R10 (ref. ^77^). Unambiguous sequences were searched on the National Center for Biotechnology Information, GenBank database using the Basic Local Alignment Search Tool (BLAST). For *COI* sequences, species-level taxonomic identities were assigned to blood meals when a host sequence was >97% similar to a sequence referenced in the database. Some unambiguous sequences did not meet this threshold. In these cases, we used the BLAST function to align and compare blood meal sequences with reference sequences obtained from tissue samples of morphologically identified species and used the same >97% homologous criterion to identify host species. For *28S ribosomal RNA* sequences, low taxonomic coverage for Annelida in the GenBank database prohibited the application of the 97% similarity criterion used for *COI*. We attributed *28S ribosomal RNA* sequences to Annelida if the most similar reference sequence to an unambiguous high-quality host sequence was derived from an annelid species. We subsequently compared *28S ribosomal RNA* blood meal sequences to reference sequences obtained from annelids collected at two Florida sites (River Styx, Alachua Co., Blue Cypress Lake, Indian River Co.). Worm specimens were captured by hand from muddy substrates where *Ur. sapphirina* mosquitoes had previously been observed. Worms were immediately placed in 95% ethanol and subsequently identified to species by M. Siddall using morphological and molecular markers.

### Statistics

The proportion of blood meals derived from vertebrate hosts and annelid hosts (detected by PCR and confirmed by sequencing) for *Ur. sapphirina* and *Ur. lowii* was compared using Fisher’s Exact Test. This analysis was performed in the software R^®^ Version 3.2.0 using the stats package. Results were considered significant if *P* *<* 0.05.

### Data availability

Sequence data generated by this study have been deposited in the National Center for Biotechnology Information GenBank database (Accession Numbers MH384533-MH384601 for annelid host sequences and Accession Numbers MH384497-MH384532 for vertebrate hosts). All other relevant data supporting the findings of this study are within the paper and its Supplementary Files. Any further data or information are available from the corresponding author upon reasonable request.

## Electronic supplementary material

Description of Additional Supplementary *Uranotaenia sapphirina*

Supplemental Movie 1
