# Supplementary material for: Identification of Uranotaenia sapphirina as a specialist of annelids broadens known mosquito host use patterns
Source: Commun Biol. 2018 Jul 12;1:92. doi: 10.1038/s42003-018-0096-5 (PMC6123777; doi:10.1038/s42003-018-0096-5)
Supplement: Supplementary file 1 — Description of Additional Supplementary Uranotaenia sapphirina [file 42003_2018_96_MOESM1_ESM.docx]

**Description of Additional Supplementary Files**

File Name: Supplementary Movie 1

Description: Video of interactions between adult female *Uranotaenia sapphirina* and earthworms. *Uranotaenia sapphirina* mosquitoes were observed and filmed feeding from annelid worms at the edge of a waterway at River Styx, Alachua Co., Florida, USA, on 10 May 2016. Toward the end of the video, the earthworms rapidly move into the substrate in response to a passing car, briefly pulling the mosquitoes downward.
